# Gender differences in oxyhemoglobin (oxy-Hb) changes during drawing interactions in romantic couples: an fNIRS study

**DOI:** 10.3389/fnbeh.2024.1476535

**Published:** 2025-01-13

**Authors:** Xinxin Huang, Limin Bai, Yantong Chen, Hongsen Cui, Lishen Wang

**Affiliations:** School of Education Science, Shaoguan University, Shaoguan, China

**Keywords:** male–female couples, fNIRS, oxyhemoglobin (oxy-Hb), real-time, drawing interactions

## Abstract

Interpersonal interaction is essential to romantic couples. Understanding how gender impacts an individual’s brain activities during intimate interaction is crucial. The present study examined gender differences in oxyhemoglobin (oxy-Hb) changes during real-time drawing interactions between members of romantic couples using non-invasive functional near-infrared spectroscopy (fNIRS). We analyzed the oxy-Hb concentrations of romantic couples engaged in interactive (i.e., chase and escape) and non-interactive (i.e., individual) drawing sessions. Our findings indicated that males (vs. females) exhibited more pronounced oxy-Hb concentrations in Broca’s area, motor area, sensorimotor cortex, and temporal lobe areas than women in an interactive drawing task, suggesting a heightened goal-oriented engagement in social interaction. Significant positive correlations were found between oxy-Hb volumes of the temporal area and the Quality of Relationship Index (QRI), underscoring the impact of interpersonal dynamics on brain function during interactive tasks. This study deepens the understanding of gender differences in neural mechanisms in social interaction tasks and provides important insights for intimacy research.

## 1 Introduction

Elucidating the dynamics of interpersonal interactions is crucial. One compelling aspect of these dynamics is how gender influences interactive behaviors and neural responses during interaction tasks. Previous studies have revealed that men and women may approach differently interactive activities (Jones et al., 2020; Huikku et al., 2022), as revealed by different communication styles (Barnett et al., 2021; Plug et al., 2021), expression of non-verbal cues (Hall et al., 2000; Thompson and Voyer, 2014), and task performance (Bartlett and Camba, 2023; Karaboga et al., 2023).

In verbal communication, women typically emphasize emotional expression and empathy (Löffler and Greitemeyer, 2023), fostering connections through supportive and inclusive dialog. In contrast, men tend to adopt a more direct and goal-oriented communication style, focusing on problem-solving and efficiency (Maliakkal and Reiter-Palmon, 2023). Furthermore, non-verbal cues also differ. Compared to men, women tend to display more facial expressions and gestures to convey emotions (Phutela, 2015). These behavioral differences extend to task performance, where women may prefer cooperative and integrative approaches, while men lean toward competitive or independent methods (Spadaro et al., 2023).

Recent advancements in social neuroscience have attracted attention to gender differences in neural activity during couple interactions (Long et al., 2022). Neurological studies have revealed distinct patterns in brain activity (Spets and Slotnick, 2021; Zhang et al., 2020) and connectivity (İçer et al., 2020; Stoica et al., 2021) between men and women during social interactions. Women typically show greater activation in the prefrontal cortex and anterior cingulate cortex, which are areas associated with empathy, emotional regulation, and social cognition (Di Tella et al., 2020; Rodríguez-Nieto et al., 2022; Taddei et al., 2023). Women also show greater activation in the insula, which is involved in emotional awareness and empathy (Wu et al., 2023). These neural patterns support the tendency for women to engage in more empathetic and emotionally expressive interactions. Men, on the other hand, often exhibit increased neural activations aligning with their tendency to endorse a more goal-oriented and less emotionally driven approach in social interactions. For example, men are found to show greater activation in brain regions involved in spatial processing (posterior cingulate) (Khaleghimoghaddam, 2024), self-referential thinking and memory (posterior cingulate) (Compère et al., 2016). Additionally, studies revealed that women have more robust interhemispheric connectivity, facilitating the integration of emotional and cognitive processes, whereas men show stronger intrahemispheric connectivity, supporting focused unilateral processing (Beltz et al., 2020). These differences may result in gender-specific behaviors in social contexts, highlighting the importance of considering these neural patterns in psychological research and practice.

Although neuroimaging studies have indicated gender differences in social interactions, their limitations do not allow for exploring of neural activity under real interpersonal interactions. Most existing studies used static measures like functional Magnetic Resonance Imaging (fMRI), which require participants to remain still, limiting the assessment of neural activity during real-time interactions. Moreover, previous studies mainly focused on gender differences in social interaction tasks but not specifically in couple interactions (Vanutelli et al., 2020), overlooking the neural mechanisms of intimate interactions. Understanding how gender differences manifest in more naturalistic settings is crucial for translating findings into practical applications. Real-time measurements of brain activity during dynamic interactions can provide more immediate and accurate insights into how gender differences unfold during a task.

Functional near-infrared spectroscopy (fNIRS) has provided a new avenue to explore gender differences at the neural level in more naturalistic settings. Importantly, fNIRS represents a non-invasive method to measure cortical activity by monitoring changes in oxyhemoglobin (oxy-Hb) and deoxyhemoglobin (deoxy-Hb) levels, allowing researchers to investigate real-time brain dynamics during interactive tasks (Huang et al., 2022). This technology is particularly suited for studying naturalistic interactions as it does not restrict movement and interaction, unlike other modalities such as fMRI. The fNIRS field has made great progress in the investigation of interpersonal interactions, including mother-infant and romantic couple interactions. The mother-infant interactions studies revealed Oxy-Hb changes in infants during different types of interactions with their mothers. For example, a study of live verbal interactions between infants and their mothers found that Oxy-Hb concentrations were higher during infants’ interactions with their mothers than during interactions with strangers (Behrendt et al., 2020). The fNIRS study of nonverbal interactions explored the relationship between cortical activation patterns and maternal sensitivity (the ability to acknowledge the infant’s needs and communications) in infants receiving maternal touch and found that the lower the mother’s sensitivity, the higher the neural response in infants’ temporal lobe regions (Mateus et al., 2021). The romantic couple’s studies mainly focused on differences in interbrain functional synchronization (IBS) between couples and other control participants (usually strangers), with the main findings showing stronger IBS in couples compared to controls. For example, Zhang et al. (2023) explored the IBS of negative emotion regulation in 30 heterosexual couples, setting strangers as a control group (Zhang et al., 2023). The results revealed that couples were able to regulate sadness more effectively, accompanied by stronger IBS. Additionally, a systematic review and meta-analysis of IBS studies in intimate relationships indicated that IBS in couples follows a similar pattern to parent–child relationships, with significant IBS brain regions concentrated in frontal, temporal, and parietal regions, implying a neural basis for social interaction (Zhao et al., 2024).

To date, the fNIRS couples’ studies suggested that couples have stronger IBS than strangers, with the main brain regions of significance including the prefrontal lobe, temporal lobe, and others. However, these studies have not analyzed gender differences between couples during intimate interactions, which is critical to understanding patterns and differences between men and women, with potential application in clinical couple therapy. Additionally, many studies have used structured tasks (Shirzadi et al., 2024) or individual activities (Sugiura et al., 2015), which do not capture the real-time interactions’ dynamic and reciprocal nature. We still know little about how gender differences manifest in more naturalistic settings.

Therefore, the present study aimed to investigate the oxy-Hb changes underlying gender differences during real-time, non-verbal drawing interactions between romantic partners by fNIRS. In this study, fNIRS was employed to measure oxy-Hb changes, aiming to explore the differences between men and women in their neural responses during various interactive drawing tasks. The present study focused on the prefrontal and temporal lobe regions, as previous research has suggested that they are the neural basis for interactive interpersonal communication (Zhao et al., 2024).

The present study also employed more naturalistic interaction tasks inspired by art therapy. Art therapy in couples holds significant potential for advancing our understanding of relational dynamics and interpersonal well-being (Van Lith, 2016), because it provides an alternative, non-verbal means of expression (e.g., interactive painting or interactive scribbling), allowing partners to convey emotions and thoughts that might be difficult to articulate verbally (Wagner and Hurst, 2018; Harris et al., 2020). Specifically, our study compared oxy-Hb changes based on the scribble interactions across different drawing conditions (i.e., chase, escape, and alone). Scribbling is a common and effective drawing style in couples’ art therapy (Revell, 1997), and is often used by clinical psychologists (Hall, 2021). In addition, the present study examined how these oxy-Hb changes correlate with relationship satisfaction. In our study, we invited participants to complete interactive (i.e., chase and escape) and non-interactive (i.e., alone) scribbling tasks. In the chase task, participants are instructed to chase their partner’s crayon with their own crayon on a piece of drawing paper. In the escape condition, participants are asked to escape from their partner’s crayon. In the alone task, participants completed scribbling by themselves.

In the present study, we hypothesized that there are significant differences in brain activation patterns between males and females in an interactive drawing task, and that these differences reflect functional properties of gender in social interaction and motor coordination tasks. We hypothesized that males exhibit higher levels of activation in brain regions associated with motor coordination and goal-directed behavior. In Broca’s area, motor area, and sensorimotor cortex, males (vs. females) showed stronger changes in oxy-Hb concentration. This hypothesis is based on research showing that males are more task-focused in body-movement control (Kuniki et al., 2021), movement planning, and goal-directed tasks (Hautala et al., 2024). Furthermore, we hypothesized that females (vs. males) exhibit higher levels of activation in brain regions associated with social cognition and emotion regulation. Females (vs. males) showed more significant brain activation in temporal lobe (specifically middle and superior temporal gyrus) and prefrontal regions (including DLPFC and frontal pole). This hypothesis was founded on the female advantage in interpreting nonverbal social cues (Obidovna, 2022) and emotional empathy (Martínez-Velázquez et al., 2020).

We additionally hypothesized that the interactive drawing task condition would further amplify this gender difference. Males and females may exhibit different activation patterns during the chase and escape tasks, reflecting gender-specific social preferences and behavioral strategies. The movement complexity in the chase task may enhance males’ activation in motor and Broca’s areas, whereas the escape task may be more involved in females’ emotion regulation processes, thus showing higher activation in temporal and prefrontal regions.

To test these hypotheses, we included Broca’s area, temporal lobe, motor area, sensorimotor cortex, DLPFC, and frontal pole as regions of interest (ROIs). The study was conducted to record brain changes across genders in an interactive drawing task by fNIRS. This study provides new perspectives for revealing gender differences in neural mechanisms in social interaction tasks and for understanding the unique contributions of gender in couple social interactions.

## 2 Methods

### 2.1 Participants

We determined our sample size by a power analysis based on prior research (Zhang et al., 2023). At least 56 participants were needed to obtain power = 0.95 (*ɑ* = 0.05). We recruited 70 participants from a university community to ensure the reliability of the experiment. All participants included were in a heterosexual relationship, right-handed, normally developed, and free from physical and mental illnesses. The exclusion criteria included being on psychotropic medication and having serious psychiatric disorders. Two participants were excluded from the fNIRS analyses due to poor signal quality. The final sample consisted of 68 participants (34 pairs of heterosexual couples; 34 females and 34 males). The age of the participants ranged between 18 and 22 years, with an average age of 20.09 years (SD = 1.19). To understand participants’ relationship experiences, we collected the number of relationships each participant had experienced and the duration of their current relationship. On average, participants had love experiences for 2.12 times 2.12 times (SD = 1.28) and had been in their current relationship for 17.58 months (SD = 13.73). All participants provided informed consent. The study adhered to the Declaration of Helsinki and was approved by the Ethics Committee of Shaoguan University (2024-YXLLSC-003).

### 2.2 Questionnaire

Participants arrived at a quiet laboratory at the agreed time, where they were briefed on the study and signed informed consent forms. Participants first filled out questionnaires collecting basic information (i.e., age, gender, number of relationships, length of current relationship) and the 6-item Quality of Relationship Index (QRI) (Patrick et al., 2007) assessing their relationship quality on a 7-point scale (1 = strongly disagree, 7 = strongly agree). The responses on the items were summed and averaged, with higher scores indicating greater relationship satisfaction (*ɑ* = 0.88).

### 2.3 Experimental procedure

Next, participants were asked to take part in the three drawing sessions. Each drawing session was repeated five times. Each couple completed two sets of 15 trials of the modified scribble chase game (Freeman, 2012). One participant wore the fNIRS device while the other did not; they switched roles for the second set. Both sets of experiments were identical. The participants were instructed to complete drawing tasks following prerecorded video-recordings throughout the experiment.

**Figure 1:**
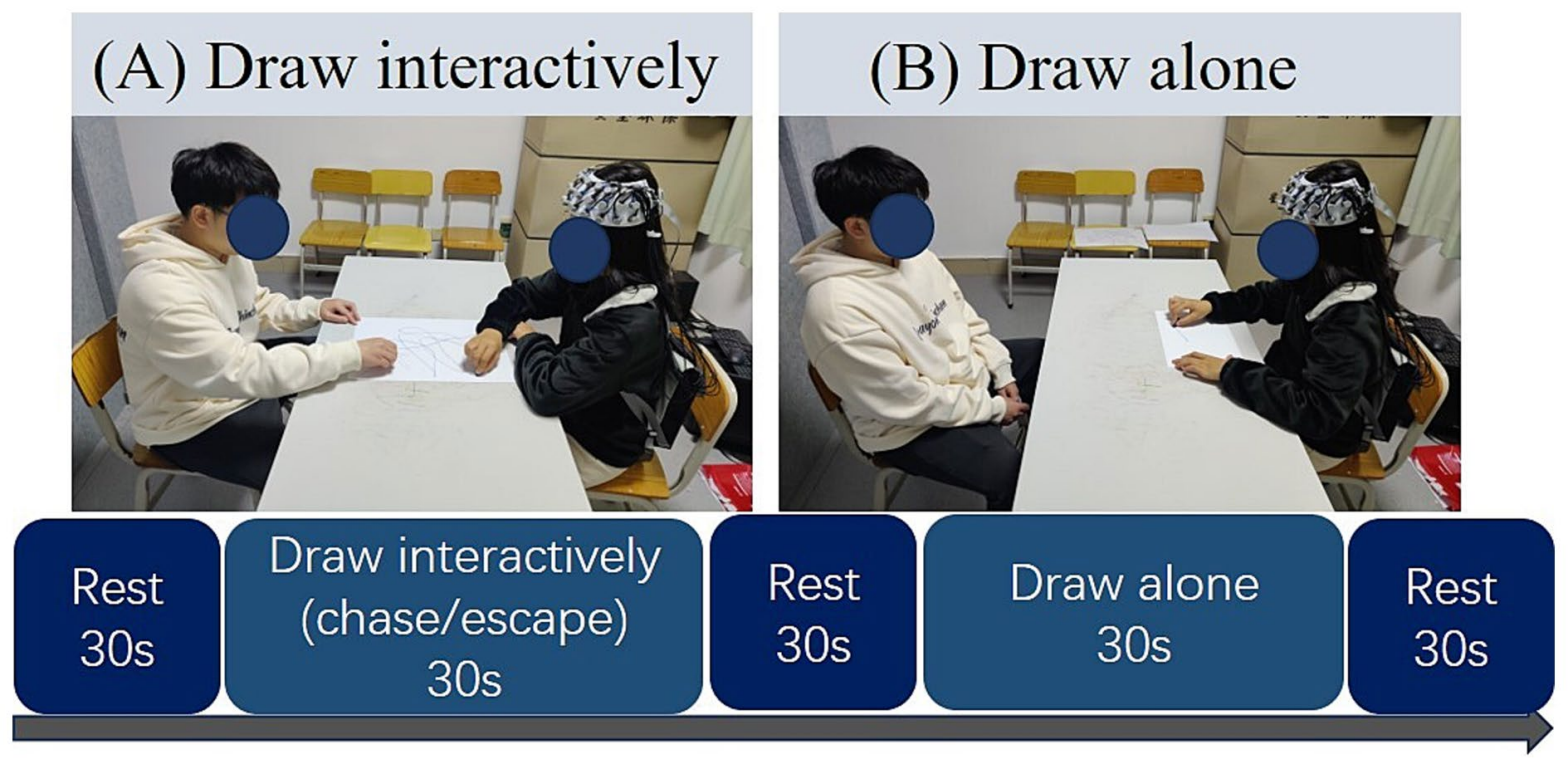
Experimental procedure. **(A)** Participant engaged in the interactive drawing tasks (chasing or escaping) with the partner; **(B)** participant engaged in the individual drawing task (draw alone condition).

The specific processes of each trial are shown in Figure 1. In each trial, the participants closed their eyes to avoid eye contact with the lover and counted silently from 1 to 5 for 30 s as rest time. Subsequently, they engaged in one of three drawing tasks for 30 s: chase (one partner tries to catch the other’s pen), escape (one partner tries to avoid the other’s pen), or draw alone (one partner draws while the other rests). Specifically, in the chase drawing, the participants and their partner each held a crayon of a different color and started at the bottom right corner of the drawing paper in front of them; when the video-recording communicated to start drawing, the participant, as the chaser, began to draw from the starting point, catching up to the partner’s pen with his or her pen on the drawing paper; and the partner, as the escapee, avoided the chaser’s crayon to keep the chaser from catching her/him. Neither subject could stop during the task; both subjects had to perform the chase and escape interactive drawing tasks for 30 s until the next recording appeared. In the escape drawing task, participants switched roles with their partners and completed the task as escapees, with the same process as the chase condition, just in different roles. After each task, participants rested by closing their eyes and counting silently from 1 to 5 for 30 s. The experimental design was counterbalanced regarding the start condition and the initial participant wearing the device.

### 2.4 fNIRS recording, preprocessing, and analysis

**Figure 2:**
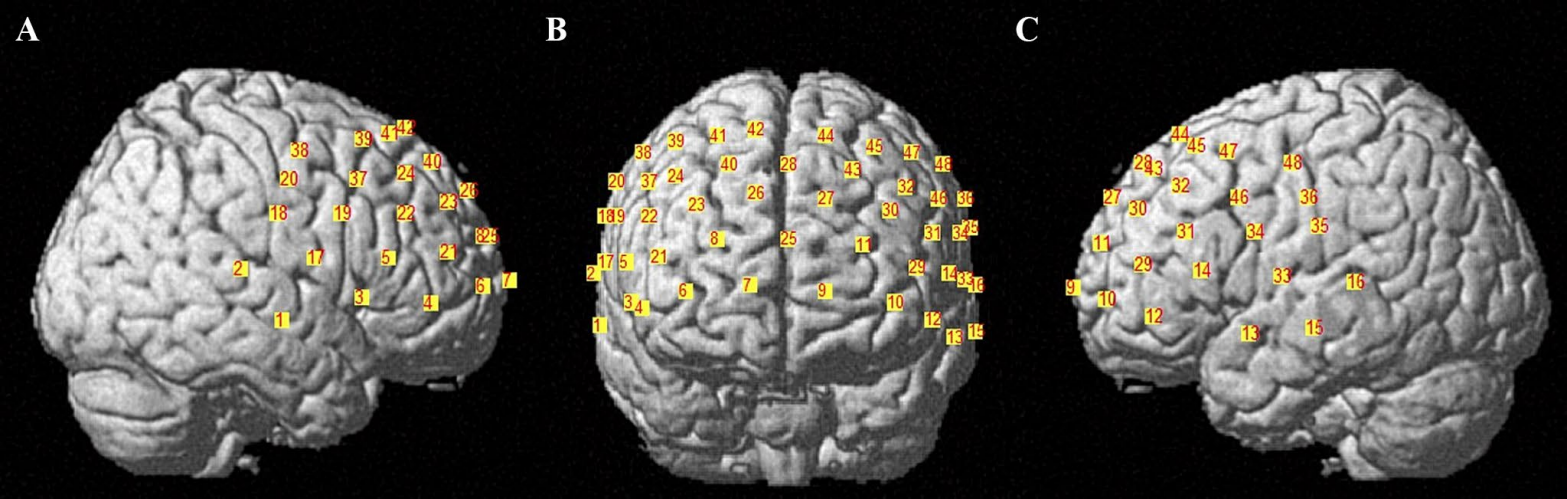
Spatial distribution of the recording channels. **(A–C)** show the fNIRS channels from the right hemisphere, frontal view and left hemisphere angles, respectively; the numbers 1–48 indicate the 48 fNIRS channels (CH 1–48).

fNIRS signals were collected using a multichannel continuous fNIRS measuring system (NirSmart II-3000A, Huichuang, China) with a sampling rate of 11 Hz, using wavelengths of 730 nm and 850 nm. The participant wore a probe cap with 15 infrared light sources and 16 detectors placed on the scalp according to the international 10–20 system (D3 corresponds to FPZ). These probes formed 48 channels (3 cm in length) covering the frontal and partial temporal cortical areas (see Figure 2). The 48 fNIRS channels were divided into six brain regions of interest (ROIs): (1) Broca’s area in the inferior frontal gyrus (left A1 and right A2; BA 44, 45); (2) the posterior part of the superior/middle temporal gyrus/temporopolar area (left B1 and right B2; BA 21, 22, 38); (3) motor-sensory areas (left C1 and right C2; including motor areas BA 6 or BA 4, and somatosensory areas BA1, 2, 3); (4) the dorsolateral prefrontal cortex (DLPFC) (left E1 and right E2; BA 9, 46); (5) the frontopolar area (left F1 and right F2; BA 10); and (6) the frontal eye fields (left F1 and right F2; BA 8). Table 1 shows the six ROIs (see Table 1). The Brodmann area distribution of each fNIRS channel is detailed in Supplementary Table 1.

**Table 1:** Distribution of ROIs.

ROIs		Channels	Brain areas	BA
A1	Left	CH12,14,29,31,46	Broca’s area in the inferior frontal gyrus	BA44,45
A2	Right	CH4,5,22,37		
B1	Left	CH13,15,16,33	Middle and Superior Temporal Gyrus, Temporopolar area	BA21,22,38
B2	Right	CH1,2,3,17,18		
C1	Left	CH34,35,36,48	Pre-Motor and Supplementary Motor Cortex, Primary Somatosensory Cortex, Primary Motor Cortex	BA1,2,3,4,6
C2	Right	CH19,20,38		
D1	Left	CH27,28,30,32,43,47	Dorsolateral prefrontal cortex (DLPFC)	BA9,46
D2	Right	CH21,23,24,26,39,40		
E1	Left	CH9,10,11,25	Frontopolar area	BA10
E2	Right	CH6,7,8		
F1	Left	CH44,45	Includes Frontal eye fields	BA8
F2	Right	CH41,42		

ROI, region of interest; BA, Brodmann area.

First, we performed a channel signal quality check to exclude channels that were judged to have poor signal quality by both the algorithms of Sappia et al. (2020) and Cui et al. (2010). In this work, we used two signal quality check functions, SQI and checkDataQuality (Sappia et al., 2020; Cui et al., 2010). They are all MATLAB-based functions based on different criteria that are independent of the specific instrument and experimental paradigm. The exclusion criterion was to remove channels with an SQI score of 1 (the best possible quality being 5) and a checkDataQuality score of bad channels, with no more than 7 channels removed for all participants.

Subsequently, we performed preprocessing and block averaging with HOMER3 (version 1.80.2) (Carius et al., 2023). Preprocessing was performed on the fNIRS data using MATLAB (MathWorks, Natick, MA, USA) using the HOMER3. We used a spline approach for motion artifact correction. We used the algorithm described by Scholkmann et al. (2020), which is implemented in the HOMER3 hmrR_MotionCorrectSpline_Nirs function. After motion artifact correction, the data were band-pass filtered with a low-pass cutoff frequency of 0.2 Hz and a high-pass cutoff frequency of 0.01 Hz. Next, the changes in optical density were converted into changes in oxy-Hb and deoxy-Hb concentrations using the modified Beer–Lambert method (path-length factor: 6.0) (Holper et al., 2009). Particularly, oxy-Hb changes were evaluated, which are more sensitive to task changes (Holtzer et al., 2019).

After calculating the values of the block average (the first 2 s of the task onset as a baseline) for each channel using HOMER3, the change in oxygenated hemoglobin (HbO) concentration of the corresponding channel was averaged according to the ROIs, and the mean value of all channels in that ROI was used to represent the change in Oxy-Hb concentration in that brain region. Finally, we fully accounted for the properties of the hemodynamic delay by averaging the data from 3 s-27 s after stimulus presentation, that is after the brain produced a response during the task, and used it to measure the activation level of the current task. Differences in brain activation levels during the chase, escape, and alone drawing tasks were further analyzed.

### 2.5 Statistical analysis

To compare differences from baseline across tasks, we performed one-sample t-tests on the means during the task period (3 s-27 s) compared against the 0 value in each ROI, and we calculated FDR-corrected *p*-values for each ROI across tasks.

The average oxy-Hb volumes were extracted from the processed fNIRS data. A 2 (gender: male/female) × 3 (condition: chase/escape/alone) mixed design ANOVA was conducted using R (version 4.3.2) and the bruce R toolkit. ANOVA was used to investigate main and interaction effects, followed by Bonferroni comparisons. Thereafter, a simple effect analysis was performed for significant interactions. ANOVA *p*-values were corrected using the false discovery rate (FDR) method (Tak and Ye, 2014; Singh and Dan, 2006). Partial eta squared (η^2^p) was used to represent the effect size of main effect and interaction of MANOVA. The thresholds for partial eta squared were as follows: 0.01–0.06, small; 0.06–0.14, moderate; > 0.14, large (Pierce et al., 2004). Pearson correlations were computed for ROIs to determine the relationship between oxy-Hb activity and QRI. Linear regression analysis was conducted to investigate the effects of oxy-Hb changes during couple interactions further. The thresholds for the correlation coefficient (r) were as follows: 0–0.1, trivial; > 0.1–0.3, weak; > 0.3–0.5, moderate; > 0.5, strong (Cohen, 2013). The significance level was set to *p* < 0.05. For each ROI, we implemented the MANOVA function by R, and tested for sphericity by using the Mauchly test integrated into the MANOVA function. When the assumption of sphericity was violated, we used the Greenhouse–Geisser correction to adjust for degrees of freedom. We then extracted *p*-values for the main effect (gender and condition) and the interaction effect (gender × condition). Finally, we applied FDR corrections to these *p*-values to control for multiple comparisons. In order to test the effect of baseline length on the experimental results, we used the same methodology for when the baseline was 5 s.

## 3 Results

### 3.1 Activations in ROIs across the drawing conditions

Results of the one-sample *t*-test revealed that eight ROIs showed positive activation in three drawing conditions, including A1 (*t* = 6.813–7.809, cohen_d = 0.862–0.872, p_adj <0.01;), A2 (t = 4.692–5.382, cohen_d = 0.569–0.653, p_adj < 0.01), B1 (*t* = 7.638–8.655, cohen_d = 0.926–1.050, p_adj <0.01), B2 (*t* = 3.020–4.737, cohen_d = 0.366–0.574, p_adj <0.01), C2 (*t* = 2.495–3.494, cohen_d = 0.303–0.424, p_adj <0.05), E1 (*t* = 2.440–2.842, cohen_d = 0.296–0.345, p_adj <0.05), and E2 (*t* = 2.824–3.386, cohen_d = 0.342–0.411, p_adj <0.01). Moreover, ROI of the C1 showed positive activation in the chase condition (*t* = 3.027, cohen_d = 0.367, p_adj <0.01) and the escape condition (*t* = 3.209, cohen_d = 0.389, p_adj <0.01), whereas there was no significant activation in the alone condition. Furthermore, ROIs of D1 (*t* = 2.191, cohen_d = 0.266, p_adj < 0.05) and D2 (*t* = 2.635, cohen_d = 0.320, p_adj <0.05) showed positive activation in the alone condition and no significant activation in the interaction conditions (chase and escape). ROIs of F1 and F2 showed no significant activation in any drawing condition (see Table 2).

**Table 2:** Activated ROIs in tasks (*n* = 68).

ROI	Chase			Escape			Alone		
	*t*	cohen_d	p_adj	*t*	cohen_d	p_adj	*t*	cohen_d	p_adj
A1	6.813	0.826	0.000	7.190	0.872	0.000	7.092	0.860	0.000
A2	4.692	0.569	0.000	4.918	0.597	0.000	5.382	0.653	0.000
B1	8.493	1.030	0.000	8.655	1.050	0.000	7.638	0.926	0.000
B2	4.737	0.574	0.000	4.178	0.506	0.000	3.020	0.366	0.009
C1	3.027	0.367	0.007	3.209	0.389	0.004	1.718	0.208	0.108
C2	3.494	0.424	0.002	3.192	0.387	0.004	2.495	0.303	0.026
D1	1.573	0.191	0.145	1.458	0.177	0.179	2.191	0.266	0.043
D2	2.114	0.256	0.051	1.835	0.223	0.095	2.635	0.320	0.021
E1	2.842	0.345	0.009	2.502	0.303	0.022	2.440	0.296	0.026
E2	2.824	0.342	0.009	3.386	0.411	0.003	3.178	0.385	0.007
F1	−0.083	−0.010	0.934	−0.361	−0.044	0.719	0.791	0.096	0.471
F2	−1.058	−0.128	0.321	−1.364	−0.165	0.193	−0.005	−0.001	0.996

ROI, region of interest; cohen_d, 0.2-small;0.5-medium;0.8-large.

### 3.2 Oxy-Hb changes in ROIs by gender and conditions

**Figure 3:**
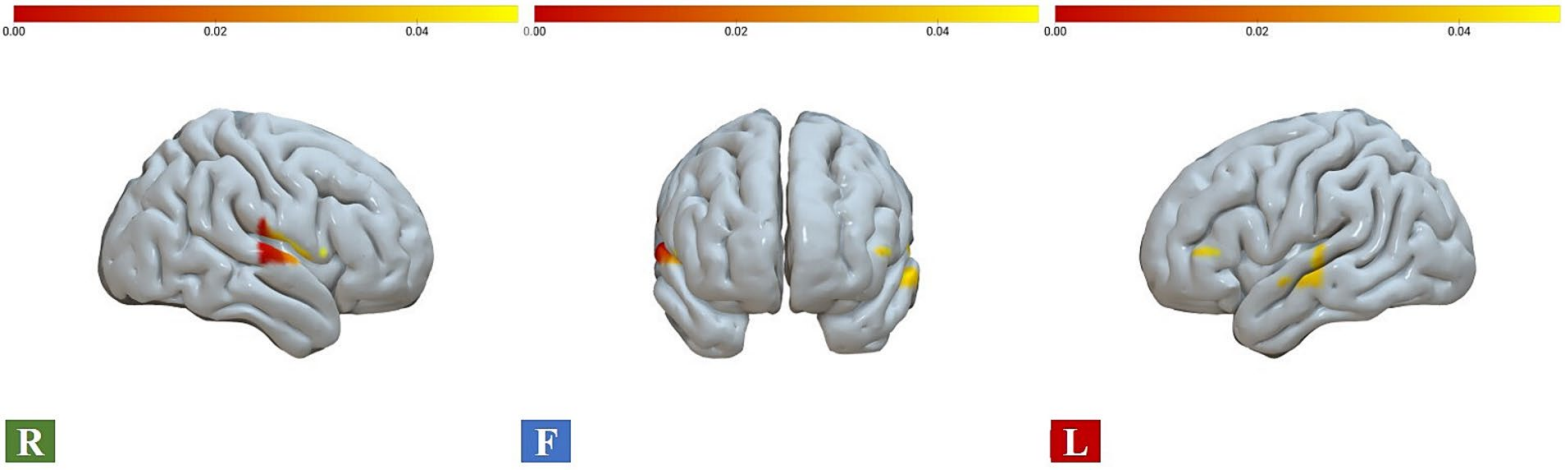
Brain activations for gender and condition interaction effects, with chromatic representation of the intensity of activation based on the *F*-values. R, right view; F, frontal view; L, left view.

**Figure 4:**
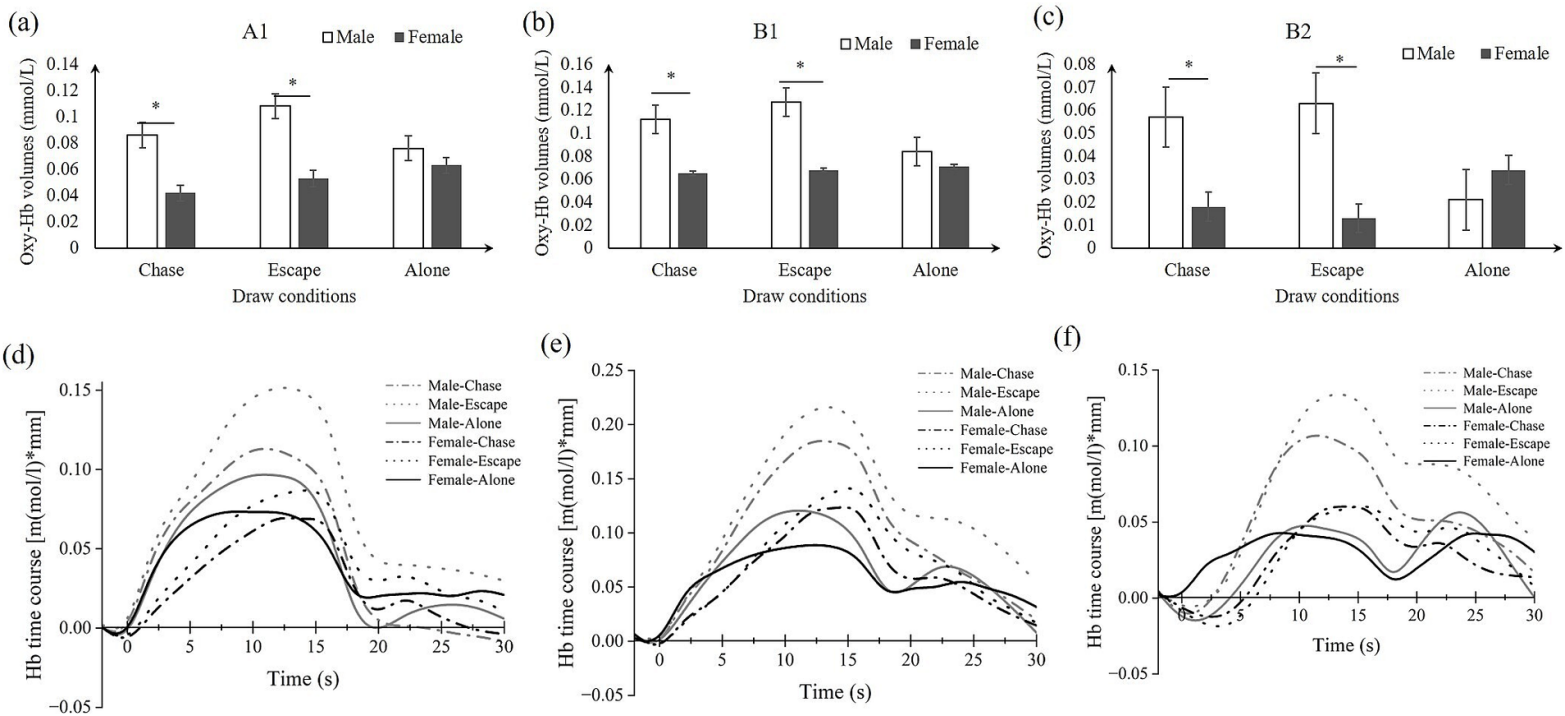
Simple effects analysis results in ROIs **(A–C)** and the time course **(D–F)**. **(A)** A1: left Broca’s area; **(B,C)** B1/B2: left/right temporal area; **(D–F)** the corresponding time course of A1/B1/B2.

As predicted, the ANOVA results showed significant interaction effects between gender and drawing conditions on three ROIs, namely A1 (*F* = 5.069, η^2^p = 0.071, FDR_*p* < 0.05), B1 (*F* = 4.885, η^2^p = 0.069, FDR_p < 0.05), and B2 (*F* = 10.604, η^2^p = 0.138, FDR_p < 0.01). Moreover, significant gender main effects were found for two ROIs, the A2 (*F* = 7.150, η^2^p = 0.098, FDR_p < 0.05) and C2 (*F* = 8.075, η^2^p = 0.109, FDR_p < 0.05). No significant condition effect was found in ROI regions (see Table 3; Figures 3, 4A–C). Time course of the significant interaction ROIs is shown in Figures 4D–F. The results for the baseline set to 5 s before the task were consistent with the results for the baseline set to 2 s before the task, illustrating the stability of the outcome (see Supplementary Table 2).

**Table 3:** Repeated measures ANOVA results for gender and conditions.

ROI	Main effect of gender			Main effect of condition			Interaction effect of gender and condition		
	*F*	FDR_p	η^2^p	*F*	FDR_p	η^2^p	*F*	FDR_p	η^2^p
A1	4.238	0.075	0.060	2.759	0.268	0.040	5.069	0.036*	0.071
A2	7.150	0.038*	0.098	0.369	0.755	0.006	3.219	0.129	0.047
B1	4.441	0.075	0.063	3.665	0.255	0.053	4.885	0.036*	0.069
B2	3.000	0.132	0.043	1.256	0.548	0.019	10.604	0.001***	0.138
C1	1.221	0.298	0.018	1.700	0.448	0.025	2.912	0.139	0.042
C2	8.075	0.036*	0.109	0.853	0.643	0.013	1.214	0.482	0.018
D1	1.927	0.204	0.028	0.439	0.755	0.007	0.638	0.636	0.010
D2	5.176	0.063	0.073	0.140	0.869	0.002	0.720	0.636	0.011
E1	0.010	0.921	0.000	1.150	0.548	0.017	0.489	0.670	0.007
E2	2.292	0.180	0.034	0.693	0.669	0.010	1.145	0.482	0.017

ROI, region of interest; FDR_p is the multiple comparison *p*-value correction using the False Discovery Rate (FDR) procedure; η^2^p: partial eta squared, 0.01–0.06, small; 0.06–0.14, moderate; > 0.14, large; A1/A2: left/right Broca’s area; B1/B2: left/right Middle and Superior Temporal Gyrus, Temporopolar area; C1/C2: left/right Pre-Motor and Supplementary Motor Cortex, Primary Somatosensory Cortex, Primary Motor Cortex; D1/D2: left/right Dorsolateral prefrontal cortex (DLPFC); E1/E2: left/right Frontopolar area; ***p < 0.001; *p < 0.05.

### 3.3 Correlation between ROIs and QRI

**Figure 5:**
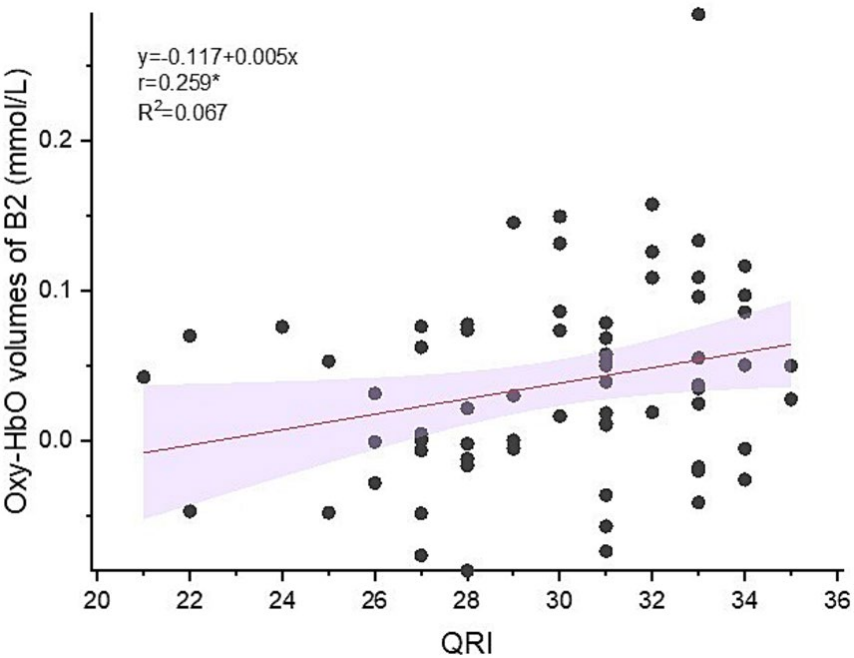
Correlation between oxy-Hb volumes in right temporal area and QRI (Quality of Relationship Index).

Significant positive correlation was found between oxy-Hb volumes in B2 and QRI (*r* = 0.259, *p* < 0.05) (see Figure 5).

## 4 Discussion

The current study investigated gender differences in brain region activation patterns during real-time drawing interactions among romantic partners, focusing on the oxy-Hb levels of interactive interactions using fNIRS. Participants, consisting of male–female couples, engaged in two types of drawing tasks: interactive (i.e., “chase,” “escape”) and non-interactive (i.e., “alone”). Oxy-Hb changes in Broca’s area, temporal lobe, motor area, sensorimotor cortex, dorsolateral prefrontal cortex (DLPFC), and frontal pole area were monitored by the fNIRS technique. Results showed that males had significantly higher oxy-Hb levels than females in the right Broca’s area, right motor area, and sensorimotor cortex. Males also exhibited significantly higher oxy-Hb levels in the left Broca’s area and bilateral temporal lobe areas than females during the chase and escape drawing conditions. Furthermore, positive correlations between oxy-Hb volumes in temporal lobe and QRI scores were observed. These findings revealed gender differences in neural mechanisms in a real-time drawing interaction task.

The results showed that males had significantly higher HbO concentrations in the left Broca’s area than females, which is consistent with our hypothesis and suggests that males may be more sensitive to the demands of action planning and strategy execution in interactive drawing tasks. Broca’s area is not only associated with language processing, but is also thought to play a key role in the comprehension of movement intentions and in movement planning for complex tasks (Papitto et al., 2024). The higher activation of males in the chase and escape condition may be related to their more competitive and goal-oriented behavior traits (Fjendbo, 2021), which may help to formulate and adapt strategies in fast-changing interactive tasks. In addition, research suggests that Broca’s area is also involved in cross-modal information integration (Yang et al., 2024), including the synergistic processing of visual and motor signals (Hickok et al., 2023). Interactive drawing tasks require participants to regulate their own movements while observing the other’s behavior, a process that may be more pronounced in males, reflecting their greater tendency to integrate movements and regulate feedback during task performance.

Furthermore, results showed that males had significantly higher HbO concentrations in bilateral temporal lobe regions than females, which is inconsistent with our expectation that females show higher activation in this region. Temporal lobe regions, particularly the middle and superior temporal gyrus, are closely associated with social cognition (Giovagnoli et al., 2021) and the processing of situational information (Oba et al., 2020). Specifically, this region has an important role in interpreting the intentions of others (Balgova et al., 2022) and processing contextually relevant social cues (Ziaei et al., 2023). In the chase and escape conditions, where the tasks placed higher demands on participants’ movement comprehension and interactive scenario modeling, the higher activation in temporal lobe regions in males may indicate a greater adaptability to the demands of these tasks. This result may be related to the specific manifestation of gender differences in social cognitive functioning. Although many studies suggest that females may be superior in emotional empathy tasks (Rochat, 2023), previous studies have focused on static tasks of face-emotion comprehension and have not addressed interpersonal tasks with specific goals (He et al., 2023). However, the tasks in the present study had explicit goals (escape or chase) and were dynamically interactive, and men’s temporal lobe activation may reflect their goal-directed behavior in understanding dynamic situations.

Moreover, the positive correlation between intimacy quality and activation levels in temporal lobe regions further supports the important role of the temporal lobe in interactive tasks. This result suggests that temporal lobe activation may not only reflect an individual’s engagement in a task, but also correlate with his or her ability to establish and maintain high-quality relationships in interpersonal interactions. Previous research indicated that couples’ neural activity strongly predicts marital satisfaction (Li et al., 2022). Our findings are consistent with that study. It may mean that changes in oxy-Hb activities during couples’ interpersonal interactions are an important physiological indicator for exploring couples’ relationship satisfaction.

In addition, the results showed significant activation of the DLPFC and frontal pole regions in interactive drawing. According to existing studies, the DLPFC and frontal pole regions are primarily involved in higher cognitive functions (Panikratova et al., 2020), including cognitive control (Yang et al., 2021), emotion regulation (Chen et al., 2023), and emotional empathy (Domínguez-Arriola et al., 2022). Particularly in tasks involving interactions with others, these regions are often thought to play an important role in supporting emotional empathy (Balconi and Angioletti, 2022) and regulating social behavior (Lin and Feng, 2024). However, the experimental results did not show significant gender differences, which is inconsistent with our hypothesis that females have higher levels of HbO activation in these regions than males. There may be differences in the demands placed on the DLPFC and frontal pole regions by the current task type. The interactive drawing task was guided by a phrase that asked subjects to chase or run away from each other’s pens as soon as possible, and the task was more focused on action planning and immediate responses to social situations than on deep processing of emotional information or long-term regulation. The DLPFC and the frontal pole regions may be more sensitive to more complex or sustained emotion-regulation tasks, whereas the dynamic interactive task in the present study may not have sufficiently mobilized these functions, resulting in a failure to reveal significant gender differences. In particular, men and women may have similar activation responses in these regions when the complexity of the task is low. Although the results do not test this hypothesis, they provided important insights suggesting that sex differences in DLPFC and frontal pole regions may depend on the nature and complexity of the task. Future studies could design more challenging or complex tasks, such as situations involving emotional conflict or long-term emotion regulation, to further explore the sex-specific functions of these regions. Meanwhile, dynamic activation patterns and sex differences in these regions may be investigated with a higher spatial resolution through the use of multimodal imaging techniques such as a combination of fMRI and fNIRS.

The present study explored brain activation patterns of males and females in an interactive drawing task, revealing the manifestation of gender differences in specific brain regions (e.g., Broca’s area, temporal lobe area, motor area, and sensorimotor cortex) and their underlying mechanisms. These results expand the understanding of the neural basis of gender in social interaction and motor planning. In particular, stronger activation in bilateral temporal lobe areas in males suggests that males may be more reliant on the integration of social context and action-related information, which provides a complementary and revised view to the traditional view that females are predominant regarding social cognition. Furthermore, the results of the present study suggest that brain activation in interactive tasks is not only influenced by gender, but may also be modulated by the type of task, contextual demands, and individual strategies. Such findings enrich the literature on the neural basis of gender and social cognition, and provide important clues for future research to delve deeper into how gender differences play a role in dynamic social tasks.

The present study has some limitations despite revealing gender-differentiated brain activation patterns in an interactive drawing task. One limitation of this study is the use of a single fNIRS device, which does not allow for monitoring brain-to-brain synchrony between partners during drawing interactions. This limits our understanding of the dynamics of mutual engagement and shared cognitive and emotional processes between partners. However, this was not the purpose of the present work. Future research should incorporate dual fNIRS systems to simultaneously monitor both partners’ neural activation, to assess interbrain synchrony and provide deeper insights into the neural mechanisms of interpersonal communication. This approach promises to enrich the theoretical framework of social neuroscience by providing a more complete picture of the neural processes that support mutual engagement and interaction in intimate relationships.

Another limitation of the present study is that the present study included participants at the age of 18, an age at which the PFC is approaching maturity levels and is already able to effectively engage in tasks such as complex cognitive control (KneŽević, 2024), emotion regulation (Liew et al., 2023), and social interaction (Li et al., 2024). However, neurological development tends to continue into the 20 years old, especially in brain regions such as the prefrontal cortex, where executive function and emotion regulation are closely linked, which does not fully mature until around 25 years old (Diamond, 2002). As the experimental tasks in this study did not involve complex interpersonal cognitive and interactional processes, and the PFC of individuals between the ages of 18 and 20 had already achieved a level of stability in some aspects, we regard that it did not affect the main results of this study. The limitation of participant age needs to be further explored in future studies, especially the effect of PFC maturation on social cognition and emotion regulation may become more pronounced as individuals enter adulthood.

Additionally, the female participants included in the current study were not observed for their menstrual cycle, which may affect women’s cognition, mood, and neural activity (Zhou et al., 2023). This limits the possibility of exploring the effects of the female menstrual cycle on couples’ interpersonal interactions, including cognitive, behavioral, and brain activity changes. Future research should consider this important factor. Moreover, data were only collected from the prefrontal and temporal lobes, and no data were collected from other brain regions, making it impossible to understand brain activity comprehensively. Therefore, future research could expand the regions of interest to explore the brain activity of couple interactions further.

## 5 Conclusion

The current fNIRS study investigated gender differences in oxy-Hb levels during couple drawing interactions. The results showed that males had higher oxy-Hb levels than females in the Broca’s area and temporal lobe during the interactive drawing process. These results contribute to a deeper understanding of gender-based neural responses during real-time interactive activities, especially in tasks that require social engagement and goal-orientation. The present study deepens the understanding of the differences in neural responses between men and women in interactive tasks.

## Data Availability

The raw data supporting the conclusions of this article will be made available by the authors, without undue reservation.
