# Supplementary material for: Gender differences in oxyhemoglobin (oxy-Hb) changes during drawing interactions in romantic couples: an fNIRS study
Source: Front Behav Neurosci. 2025 Jan 13;18:1476535. doi: 10.3389/fnbeh.2024.1476535 (PMC11769947; doi:10.3389/fnbeh.2024.1476535)
Supplement: Supplementary file 1 [file Data_Sheet_1.PDF]

Supplementary Material

1 Supplementary Tables

Table 1. MNI and Brodmann areas related to fNIRS channels.

Channel	MNI (x, y, z)	Brodmann Area (Brain Region)	Percentage of Overlap
CH1 (S1-D1)	69.078, -12.188, -5.904	21 - Middle Temporal gyrus	0.805
		22 - Superior Temporal Gyrus	0.195
CH2 (S1-D6)	71.400, -27.082, 11.832	2 - Primary Somatosensory Cortex	0.012
		21 - Middle Temporal gyrus	0.103
		22 - Superior Temporal Gyrus	0.879
		48 - Retrosubicular area	0.006
CH3 (S2-D1)	59.235, 16.768, 1.141	6 - Pre-Motor and Supplementary Motor Cortex	0.048
		38 - Temporopolar area	0.4237
		44 - pars opercularis_ part of Broca's area	0.058
		45 - pars triangularis Broca's area	0.071
		48 - Retrosubicular area	0.4
CH4 (S2-D2)	54.096, 44.496, 0.631	45 - pars triangularis Broca's area	0.457

		46 - Dorsolateral prefrontal cortex	0.543
CH5 (S2-D7)	59.467, 29.492, 15.871	44 - pars opercularis_ part of Broca's area	0.065
		45 - pars triangularis Broca's area	0.935
CH6 (S3-D2)	38.368, 63.452, 5.227	10 - Frontopolar area	0.830
		11 - Orbitofrontal area	0.093
		46 - Dorsolateral prefrontal cortex	0.078
CH7 (S3-D3)	14.243, 72.998, 8.398	10 - Frontopolar area	1
CH8 (S3-D8)	25.779, 64.115, 23.978	9 - Dorsolateral prefrontal cortex	0.019
		10 - Frontopolar area	0.761
		46 - Dorsolateral prefrontal cortex	0.220
CH9 (S4-D3)	-15.037, 73.097, 7.045	10 - Frontopolar area	0.967
		11 - Orbitofrontal area	0.033
CH10 (S4-D4)	-41.007, 62.253, 0.949	10 - Frontopolar area	0.676
		46 - Dorsolateral prefrontal cortex	0.324
CH11 (S4-D9)	-28.425, 62.695, 22.046	10 - Frontopolar area	0.512
		46 - Dorsolateral prefrontal cortex	0.488
CH12 (S5-D4)	-55.457, 41.980, -3.865	45 - pars triangularis Broca's area	0.509

		46 - Dorsolateral prefrontal cortex	0.396
		47 - Inferior prefrontal gyrus	0.094
CH13 (S5-D5)	-62.539, 6.834, -9.078	21 - Middle Temporal gyrus	0.469
		38 - Temporopolar area	0.447
		48 - Retrosubicular area	0.084
CH14 (S5-D10)	-61.036, 23.610, 12.478	6 - Pre-Motor and Supplementary Motor Cortex	0.028
		44 - pars opercularis_ part of Broca's area	0.313
		45 - pars triangularis Broca's area	0.56
		48 - Retrosubicular area	0.101
CH15 (S6-D5)	-71.255, -18.293, -9.431	20 - Inferior Temporal gyrus	0.013
		21 - Middle Temporal gyrus	0.930
		22 - Superior Temporal Gyrus	0.057
CH16 (S6-D11)	-71.922, -33.954, 7.951	21 - Middle Temporal gyrus	0.179
		22 - Superior Temporal Gyrus	0.821
CH17 (S7-D1)	66.569, 0.761, 15.568	6 - Pre-Motor and Supplementary Motor Cortex	0.283
		22 - Superior Temporal Gyrus	0.089
		43 - Subcentral area	0.406
		48 - Retrosubicular area	0.222

CH18 (S7-D6)	68.372, -14.129, 30.974	1 - Primary Somatosensory Cortex	0.137
		2 - Primary Somatosensory Cortex	0.357
		43 - Subcentral area	0.506
CH19 (S7-D7)	62.995, 9.827, 30.594	4 - Primary Motor Cortex	0.039
		6 - Pre-Motor and Supplementary Motor Cortex	0.629
		43 - Subcentral area	0.039
		44 - pars opercularis_ part of Broca's area	0.293
CH20 (S7-D12)	62.633, -9.671, 43.314	1 - Primary Somatosensory Cortex	0.190
		3 - Primary Somatosensory Cortex	0.218
		4 - Primary Motor Cortex	0.296
		6 - Pre-Motor and Supplementary Motor Cortex	0.155
		43 - Subcentral area	0.141
CH21 (S8-D2)	45.905, 50.151, 18.427	45 - pars triangularis Broca's area	0.307
		46 - Dorsolateral prefrontal cortex	0.693
CH22 (S8-D7)	50.711, 34.608, 32.429	44 - pars opercularis_ part of Broca's area	0.090
		45 - pars triangularis Broca's area	0.808
		46 - Dorsolateral prefrontal cortex	0.102
CH23 (S8-D8)	34.291, 49.297, 35.141	9 - Dorsolateral prefrontal cortex	0.377

		46 - Dorsolateral prefrontal cortex	0.623
CH24 (S8-D13)	41.190, 33.052, 45.806	9 - Dorsolateral prefrontal cortex	0.842
		44 - pars opercularis_ part of Broca's area	0.032
		45 - pars triangularis Broca's area	0.054
		46 - Dorsolateral prefrontal cortex	0.072
CH25 (S9-D3)	-1.219, 64.658, 24.060	10 - Frontopolar area	1
CH26 (S9-D8)	12.751, 58.709, 39.673	9 - Dorsolateral prefrontal cortex	0.881
		10 - Frontopolar area	0.120
CH27 (S9-D9)	-13.901, 58.599, 39.338	9 - Dorsolateral prefrontal cortex	0.877
		10 - Frontopolar area	0.098
		46 - Dorsolateral prefrontal cortex	0.025
CH28 (S9-D14)	-2.323, 46.909, 50.715	8 - Includes Frontal eye fields	0.307
		9 - Dorsolateral prefrontal cortex	0.693
CH29 (S10-D4)	-49.171, 47.315, 14.950	45 - pars triangularis Broca's area	0.5780
		46 - Dorsolateral prefrontal cortex	0.422
CH30 (S10-D9)	-37.759, 48.262, 32.857	9 - Dorsolateral prefrontal cortex	0.099
		45 - pars triangularis Broca's area	0.121
		46 - Dorsolateral prefrontal cortex	0.780

CH31 (S10-D10)	-55.244, 30.450, 27.438	44 - pars opercularis_ part of Broca's area	0.133
		45 - pars triangularis Broca's area	0.867
CH32 (S10-D15)	-44.876, 31.592, 42.658	9 - Dorsolateral prefrontal cortex	0.441
		44 - pars opercularis_ part of Broca's area	0.225
		45 - pars triangularis Broca's area	0.225
		46 - Dorsolateral prefrontal cortex	0.108
CH33 (S11-D5)	-67.886, -5.171, 9.921	6 - Pre-Motor and Supplementary Motor Cortex	0.023
		22 - Superior Temporal Gyrus	0.442
		43 - Subcentral area	0.204
		48 - Retrosubicular area	0.331
CH34 (S11-D10)	-65.215, 5.784, 26.201	4 - Primary Motor Cortex	0.021
		6 - Pre-Motor and Supplementary Motor Cortex	0.567
		43 - Subcentral area	0.344
		44 - pars opercularis_ part of Broca's area	0.067
CH35 (S11-D11)	-69.471, -19.510, 27.053	1 - Primary Somatosensory Cortex	0.016
		2 - Primary Somatosensory Cortex	0.557
		22 - Superior Temporal Gyrus	0.104
		42 - Primary and Auditory Association Cortex	0.003

		43 - Subcentral area	0.207
		48 - Retrosubicular area	0.113
CH36 (S11-D16)	-65.540, -14.600, 39.308	1 - Primary Somatosensory Cortex	0.354
		2 - Primary Somatosensory Cortex	0.175
		3 - Primary Somatosensory Cortex	0.193
		4 - Primary Motor Cortex	0.025
		43 - Subcentral area	0.253
CH37 (S12-D7)	53.678, 15.314, 44.390	6 - Pre-Motor and Supplementary Motor Cortex	0.235
		9 - Dorsolateral prefrontal cortex	0.384
		44 - pars opercularis_ part of Broca's area	0.381
CH38 (S12-D12)	53.020, -6.226, 55.067	4 - Primary Motor Cortex	0.351
		6 - Pre-Motor and Supplementary Motor Cortex	0.649
CH39 (S12-D13)	42.854, 16.766, 56.627	6 - Pre-Motor and Supplementary Motor Cortex	0.127
		8 - Includes Frontal eye fields	0.131
		9 - Dorsolateral prefrontal cortex	0.742
CH40 (S13-D8)	21.201, 43.716, 50.303	8 - Includes Frontal eye fields	0.117
		9 - Dorsolateral prefrontal cortex	0.883
CH41 (S13-D13)	27.230, 27.605, 58.695	8 - Includes Frontal eye fields	0.954

		9 - Dorsolateral prefrontal cortex	0.046
CH42 (S13-D14)	11.586, 33.676, 61.483	8 - Includes Frontal eye fields	1
CH43 (S14-D9)	-24.808, 42.433, 48.690	8 - Includes Frontal eye fields	0.036
		9 - Dorsolateral prefrontal cortex	0.964
CH44 (S14-D14)	-14.019, 32.636, 60.700	8 - Includes Frontal eye fields	0.985
		9 - Dorsolateral prefrontal cortex	0.015
CH45 (S14-D15)	-33.398, 26.161, 57.267	8 - Includes Frontal eye fields	0.565
		9 - Dorsolateral prefrontal cortex	0.435
CH46 (S15-D10)	-57.258, 11.408, 37.888	6 - Pre-Motor and Supplementary Motor Cortex	0.528
		9 - Dorsolateral prefrontal cortex	0.026
		44 - pars opercularis_ part of Broca's area	0.446
CH47 (S15-D15)	-47.267, 14.526, 54.181	6 - Pre-Motor and Supplementary Motor Cortex	0.354
		9 - Dorsolateral prefrontal cortex	0.646
CH48 (S15-D16)	-57.448, -9.533, 50.728	3 - Primary Somatosensory Cortex	0.208
		4 - Primary Motor Cortex	0.392
		6 - Pre-Motor and Supplementary Motor Cortex	0.4

Table2: Repeated measures ANOVA results for gender and conditions (baseline 5s)

ROI	Main effect of gender			Main effect of condition			Interaction effect of gender and condition		
	F	*FDR_p*	η^2p	*F*	*FDR_p*	η^2p	*F*	*FDR_p*	η^2p
A1	4.320	0.079	0.061	2.288	0.367	0.034	**5.393**	**0.022**	**0.076**
A2	**7.070**	**0.039**	**0.097**	0.537	0.703	0.008	3.654	0.069	0.052
B1	4.125	0.079	0.059	2.557	0.367	0.037	**5.828**	**0.022**	**0.081**
B2	2.517	0.171	0.037	0.982	0.646	0.015	**11.842**	**0.000**	**0.152**
C1	0.967	0.359	0.014	1.289	0.646	0.019	3.643	0.069	0.052
C2	**8.037**	**0.036**	**0.109**	0.554	0.706	0.008	1.294	0.476	0.019
D1	1.929	0.203	0.028	0.411	0.724	0.006	0.834	0.476	0.012
D2	5.260	0.060	0.074	0.198	0.820	0.003	0.878	0.476	0.013
E1	0.016	0.899	0.000	1.115	0.646	0.017	0.838	0.476	0.013
E2	2.377	0.171	0.035	0.620	0.703	0.009	1.594	0.414	0.024

Note: ROI: region of interest; FDR_p is the multiple comparison p-value correction using the False Discovery Rate (FDR) procedure; η^2p: partial eta squared, 0.01–0.06, small; 0.06–0.14, moderate; > 0.14, large; A1/A2: left/right Broca's area; B1/B2: left/right Middle and Superior Temporal Gyrus, Temporopolar area; C1/C2: left/right Pre-Motor and Supplementary Motor Cortex, Primary Somatosensory

Cortex, Primary Motor Cortex; D1/D2: left/right Dorsolateral prefrontal cortex (DLPFC); E1/E2: left/right Frontopolar area; ***p<0.001; **p < 0.01; *p < 0.05.
